# Supplementary material for: The design and implementation of restraint devices for the injection of pathogenic microorganisms into Galleria mellonella
Source: PLoS One. 2020 Jul 30;15(7):e0230767. doi: 10.1371/journal.pone.0230767 (PMC7392296; doi:10.1371/journal.pone.0230767)
Supplement: S1 Table — (DOCX) [file pone.0230767.s003.docx]

| Brand | Tip type | Catalog # | Cut sites (from the widest end) |
| --- | --- | --- | --- |
| Vistalab | P250 | 4058-2000 | 1 cut: 2.5 cm |
| Vistalab | P1250 | 4226 | 2 cuts: 4.1 cm, 7.1 cm |
| Fisher Science | P1250 | 02-707-400 | 2 cuts: 4.1 cm, 6.6 cm |
| Thermo Fisher | P1250 | 2789-HR | 2 cuts: 3.8 cm, 6.3 cm |
| Denville Scientific | P1250 | 1158U31 | 2 cuts: 5.0 cm, 7.1 cm |
